# Battlefield Acupuncture (BFA) for Management of Chronic Pain in an Outpatient Setting

**DOI:** 10.1093/geroni/igaf122.3719

**Published:** 2025-12-31

**Authors:** Ana Maria Pasatiempo, Rachel Ceria, Stephanie Hicks, Jessika Dayrit

**Affiliations:** VA Pacific Islands Health Care System, Department of Veterans Affairs, Alaska, United States; VA Pacific Islands Health Care System, Honolulu, Hawaii, United States; VA Pacific Islands Health Care System, Honolulu, Hawaii, United States; VA Pacific Islands Health Care System, Honolulu, Hawaii, United States

## Abstract

**Background:**

Battlefield Acupuncture (BFA) is a non-pharmacological auricular acupuncture technique used to manage chronic pain. This study evaluated the short-term and longitudinal effectiveness of BFA among veterans receiving care in the Veteran Health Administration (VHA) community clinic.

**Methods:**

Prospective pre-post interventional study was conducted at the VHA Guam Community-Based Outpatient Clinic (2018-2019). Fifty-four veterans with chronic pain received BFA delivered by trained clinicians. Pain intensity was assessed immediately before and after each session using the Defense and Veterans Pain Rating Scale (DVPRS). Final analytic sample included19 participants who completed at least five sessions. Short-term effects were examined by comparing pre-post pain scores for each session; cumulative change was evaluated by comparing pre-session scores from first and fifth visits. Non-parametric tests accounted for the ordinal nature of pain ratings.

**Results:**

Significant reductions in pain were observed after each session (mean= 2.68, SD = 1.73). Subsequent treatments also demonstrated significant pain reduction (2nd session, mean=2.05 with SD = 2.09, 3rd session, mean=2.16 with SD = 1.92, 4th session, mean =1.94 with SD = 1.84, 5th session with mean 1.58 with SD = 1.92). However, cumulative improvement across sessions was not statistically significant (χ^2^(4) = 3.40, p = 0.49).

**Conclusion:**

Older Veterans often rely on opioids for chronic pain, which may lead to dependency and side effects. BFA produced short-term effectiveness in reducing chronic pain, with significant relief after individual sessions. However, sustained improvement over multiple treatments was not observed. Limitations include small sample size, lack of a control group, and potential confounders. Larger controlled studies are warranted to assess long-term efficacy.

